# Evidence for oxidative stress in the developing cerebellum of the rat after chronic mild carbon monoxide exposure (0.0025% in air)

**DOI:** 10.1186/1471-2202-10-53

**Published:** 2009-05-27

**Authors:** Ivan A Lopez, Dora Acuna, Luis Beltran-Parrazal, Ivan E Lopez, Abhimanyu Amarnani, Max Cortes, John Edmond

**Affiliations:** 1 Department of Surgery (Division of Head and Neck), 31-25 Rehabilitation Center, 1000 Veteran Avenue, David Geffen School of Medicine at UCLA, Los Angeles, CA 90095, USA; 2 Mental Retardation Research Center, Neuroscience Research Building, Room 260C, 635 Charles E Young Drive South, David Geffen School of Medicine at UCLA, Los Angeles, CA 90095-7332, USA

## Abstract

**Background:**

The present study was designed to test the hypothesis that chronic very mild prenatal carbon monoxide (CO) exposure (25 parts per million) subverts the normal development of the rat cerebellar cortex. Studies at this chronic low CO exposure over the earliest periods of mammalian development have not been performed to date. Pregnant rats were exposed chronically to CO from gestational day E5 to E20. In the postnatal period, rat pups were grouped as follows: Group A: prenatal exposure to CO only; group B: prenatal exposure to CO then exposed to CO from postnatal day 5 (P5) to P20; group C: postnatal exposure only, from P5 to P20, and group D, controls (air without CO). At P20, immunocytochemical analyses of oxidative stress markers, and structural and functional proteins were assessed in the cerebellar cortex of the four groups. Quantitative real time PCR assays were performed for inducible (iNOS), neuronal (nNOS), and endothelial (eNOS) nitric oxide synthases.

**Results:**

Superoxide dismutase-1 (SOD1), SOD2, and hemeoxygenase-1 (HO-1) immunoreactivity increased in cells of the cerebellar cortex of CO-exposed pups. INOS and nitrotyrosine immunoreactivity also increased in blood vessels and Purkinje cells (PCs) of pups from group-A, B and C. By contrast, nNOS immunoreactivity decreased in PCs from group-B. Endothelial NOS immunoreactivity showed no changes in any CO-exposed group. The mRNA levels for iNOS were significantly up-regulated in the cerebellum of rats from group B; however, mRNA levels for nNOS and eNOS remained relatively unchanged in groups A, B and C. Ferritin-H immunoreactivity increased in group-B. Immunocytochemistry for neurofilaments (structural protein), synapsin-1 (functional protein), and glutamic acid decarboxylase (the enzyme responsible for the synthesis of the inhibitory neurotransmitter GABA), were decreased in groups A and B. Immunoreactivity for two calcium binding proteins, parvalbumin and calbindin, remained unchanged. The immunoreactivity of the astrocytic marker GFAP increased after prenatal exposure.

**Conclusion:**

We conclude that exogenously supplied CO during the prenatal period promotes oxidative stress as indicated by the up-regulation of SOD-1, SOD-2, HO-1, Ferritin-H, and iNOS with increased nitrotyrosine in the rat cerebella suggesting that deleterious and protective mechanisms were activated. These changes correlate with reductions of proteins important to cerebellar function: pre-synaptic terminals proteins (synapsin-1), proteins for the maintenance of neuronal size, shape and axonal quality (neurofilaments) and protein involved in GABAergic neurotransmission (GAD). Increased GFAP immunoreactivity after prenatal CO-exposure suggests a glial mediated response to the constant presence of CO. There were differential responses to prenatal vs. postnatal CO exposure: Prenatal exposure seems to be more damaging; a feature exemplified by the persistence of markers indicating oxidative stress in pups at P20, following prenatal only CO-exposure. The continuation of this cellular environment up to day 20 after CO exposure suggests the condition is chronic. Postnatal exposure without prenatal exposure shows the least impact, whereas prenatal followed by postnatal exposure exhibits the most pronounced outcome among the groups.

## Background

Carbon monoxide (CO) is a colorless, tasteless and odorless gas product of the incomplete combustion of carbon-based fuels and other carbonaceous substances [1]. From a public health perspective, CO toxicity is the most common type of accidental poisoning in the United States and the cause of more that 50% of fatal poisonings in many industrial countries [2,3]. CO has been also identified as an important endogenous biological messenger in the brain [4,5], and it is a major component in the regulation of the cerebro-vascular circulation in newborns [6]. The central nervous system (CNS) is the most sensitive tissue to CO, and usually suffers the greatest lasting damage from CO poisoning [7].

There is an increase in clinical awareness that a link exists between chronic exposure to CO and the etiology of vascular or cardiovascular diseases [1,8-10]. Several studies on humans (prenatal and postnatal periods) provide data that CO and cigarette smoke affects normal growth and development [11-14]. Adverse effects of prenatal tobacco smoke exposure on the developing brain stem in humans have also been reported [15]. CO readily crosses the placenta and binds to fetal hemoglobin [12,16]. Gestational exposure to CO impairs vascular reactivity in rat offspring at different stages of neurogenesis [17]. The developing CNS and peripheral nervous system are extremely susceptible to the reduction of oxygen availability produced by CO exposure [18], and both neurobehavioral and neurochemical alterations have been found in male rat offspring exposed to low levels of CO during gestation [17,19-21]. Adverse effects have been found in the developing peripheral tissues of a prenatal exposure model that simulates the CO exposure conditions observed in human cigarette smokers [13].

As reviewed by Benagiano et al., [22,23], numerous reports suggest that intrauterine and neonatal exposure to mild CO concentrations affects the course of CNS development. Studies have been done with chronic exposure of pregnant rats to CO at 75–150 ppm; concentrations below those associated with gross malformations or overt neurotoxic effects in the offspring [22,23]. However, mild CO exposure induced long-lasting consequences in the prenatal developing nervous system, and it is possibly implicated in the pathogenesis of persistent permanent neurological disorders detectable in adulthood [24-27]. Various biochemical and functional alterations have been found postnatally in the rat CNS after such relatively mild prenatal exposure to CO, including: change in the content of some neurotransmitters in the brain stem and cerebellum [28-30], dysfunction of the dopaminergic mesolimbic system [19], impairment of the maintenance of long term potentiation associated with a decreased activity of the enzymes heme oxygenase-2 (HO-2) and nitric oxide synthase (NOS) in the hippocampus [12].

The cerebellum is a highly organized structure in which Purkinje cells (PCs) are the sole output of the cerebellar cortex [31-34], and it is one of the first structures of the brain to differentiate, however, it achieves its mature configuration many months after birth [35,36]. For this reason the cerebellum is especially vulnerable to developmental irregularities [36,37]. Exposure to CO induces the formation of COHb and consequently a decrease of the tissue oxygen supply (hypoxia) [22,23]. The developing cerebellum is highly susceptible to prenatal CO exposure at low concentrations [28,29]. Studies on animal models have shown that PCs are vulnerable to ischemia, hypoxia, excitotoxicity, oxidative stress and exposure to different types of chemicals [37]. It has been well documented that prenatal CO exposure has selective effects on the different cell types [22]. Benagiano et al., found that Purkinje and Golgi neurons did not appear to be susceptible, unlike the postnatally generated stellate and basket neurons [22]. For these reasons it is possible that very mild chronic exposure to CO may affect cerebellar neurons.

We have previously studied the effect of chronic mild CO exposures in the concentration range 12–25 ppm (concentrations at or below upper limits established by several regulatory agencies) in the developing auditory system of the rat [38-42]. The present study was designed to test the hypothesis that chronic very mild prenatal CO exposure (25 ppm) subverts the normal development of the cerebellar cortex. Pregnant rats at E5 were exposed to CO at 25 parts per million (ppm). Rat cerebella were examined by immunocytochemical methods at postnatal day 20 (P20). Specifically we examined the immunolocalization of oxidative stress proteins as well as proteins relevant to cerebellar function and development. Oxidative stress markers included superoxide dismutase-1 (SOD-1) and SOD-2, heme-oxygenase-1 (HO-1), and HO-2, inducible nitric oxide synthase (iNOS), neuronal NOS (nNOS) and endothelial NOS (eNOS) and Ferritin. The presence of nitrotyrosine (NT) was also investigated. Neurons were identified using pan-neurofilaments, and the presence of synaptic terminals was studied using antibodies against synapsin-1. Glial cells were identified using glial fibrillary acidic protein (GFAP). Two calcium binding proteins, parvalbumin (PV) and calbindin-D28K (CB) were also used to identify neurons of the cerebella and their terminals. To determine whether the GABAergic system of the cerebella was altered after CO exposure, we used antibodies against glutamic acid decarboxylase (GAD-67). Preliminary portions of these data have been presented in abstract form [43,44].

## Methods

### Animal use

The Chancellor's Animal Subject Protection Committee at the University of California, Los Angeles, approved the research protocol for the use of animal subjects in this study. Animals were handled and cared for in accordance with the Animal Welfare Act and in strict compliance with the National Institutes of Health Guidelines.

### CO exposure

Pregnant Sprague-Dawley rats (second or third litter experience) were obtained from the supplier (Charles River) at 2 days of gestation. Cages containing the pregnant rats were enclosed by gas tight chambers that were constructed of clear plastic to contain 130 liters of air and through which gas flowed at controlled rates to obtain the desired CO exposure conditions. The chambers were removed from the exposure system daily to allow for the maintenance of the animals [40,43]. The gas delivered to the CO-chamber contains a combination of 25 ppm CO in 22% oxygen with the balance of the gas as nitrogen and carbon dioxide at appropriate levels. Gas was administered continuously for 10–18 hours during the day and the gas supply-system and chambers removed at night (approximately 10 hrs). Oxygen concentrations have been shown to never fall below 21.5% in the air breathed by the animals. The same gas mixture but without CO was delivered to the chambers containing the control animals [42].

### Animal groups

Group A consists of rat pups born from pregnant rats exposed to CO (25 ppm) from E5 to E20. Group B, consist of rat pups exposed as group A and then exposed again to CO from P5 to P20. Group C consists of rat pups from mothers not exposed prenatal to CO (air without CO only), but exposed to CO in the postnatal period from P5 to P20. Group D are the controls that were never exposed to CO at any time. For the rats to be exposed to CO in air, or air without CO, postnatal day 0 (P0) is the calendar day on which the rat pups were born. There was no difference in the body weight of rat pups (from comparable litter sizes) between CO exposed and air-only exposed rat pups. This experiment was repeated three times.

### Tissue processing

On P20, CO exposed rat pups and age-matched controls were over anesthetized with halothane, and then decapitated. The whole brain was dissected from the head, and then immersed in 4% sodium phosphate buffered (0.11 M, pH 7.4) paraformaldehyde solution for 8–12 hours. Thereafter the whole brain was immersed in 30% sucrose dissolved in buffered phosphate saline solution (PBS), for 5–7 days. The cerebella was separated from the brain and embedded with O.C.T. compound (Tissue-Tek, Ted Pella), and 20 μm thick sagittal sections were obtained (Cryostat, model Microm HN505E). Tissue sections were mounted on Superfrost Plus glass slides (Fisher Scientific) and stored at -80°C until their use.

### Immunocytochemistry

Cerebella from each group were dehydrated, embedded in Paraffin Paraplast (Fisher Scientific), and 20 μm thick sections were obtained. Before immunocytochemistry paraffin was removed and tissue sections were rehydrated. Sections were incubated at room temperature for 10 minutes with 3% hydrogen peroxide (diluted in 100% methanol), then washed three times with PBS and incubated one hour with a blocking solution containing 5% normal goat or horse serum and 1% Triton X-100 diluted in buffered PBS. Next, the solution was removed and the primary polyclonal or monoclonal antibody was applied to the tissue sections at dilutions indicated in Table 1. Tissue sections were incubated in a humid chamber at 4°C for 48 hrs. Tissue sections were then incubated with the biotinylated goat anti-rabbit or goat anti-mouse secondary antibody (1:250) for 1 hour at room temperature (Elite kit, Vector Labs). Sections were then incubated with the avidin-biotin-peroxidase solution ABC (Elite kit, Vector Labs) for 1 hour at room temperature. The antigen-antibody reaction was visualized using diaminobenzidine (DAB) as the chromogen (Vector labs Kit). The reaction was halted after 2–4 minutes with distilled water (15 minutes × 3). The sections were coversliped with Vector aqueous mounting media for light microscopic examination (Vector Labs).

**Table 1:** Antibodies used in the present study, source, dilution, type, species reactivity, immunogen, negative and positive controls used.

**Antibody/Source**	**Dilution/antibody type**	Species reactivity/immunogen ***	**Negative control**	**Positive control/cellular location**
Pan neurofilament/Zymed	1:500/mouse monoclonal	Rat, mouse, human/human cocktail neurofilament low, middle and high molecular weight peptides	No antibody on ICC	Mouse, rat human, cerebellar cortex*/neuron perikarya and axons
Synapsin-1/Chemicon	1:1000/rabbit polyclonal	Rat, bovine, human/synapsin I (Ia and Ib) bovine brain	No antibody on ICC	Mouse, rat cerebellar cortex*/nerve terminals
Calbindin/Sigma	1:1000/mouse monoclonal	Rat, mouse, human, goat/bovine kidney – calbindin D-28k	No antibody on ICC	Mouse, rat cerebellar cortex*/Purkinje cell arborization
Parvalbumin/Chemicon	1:1000/mouse monoclonal	Rat, human, bovine/Parvalbumin purified from frog muscle	No antibody on ICC	Purkinje cell arborization, basket cells mice and human cerebella
SOD-1/Santa Cruz	1:500/rabbit polyclonal	Rat, mouse, human/aa 1–154 full length human SOD-1	Antibody absorption with peptide on ICC	Rat* and mice** intestine/luminal epithelial cells
SOD2/Abcam	1:1000/rabbit polyclonal	Drosophila and several vertebrates/full length rat SOD-2 protein	No antibody on ICC	Mouse and rat cerebellar cortex and brain stem/neuronal soma
HO-1/Santa Cruz	1:500/Goat polyclonal	Rat, mouse, human/peptide mapping C-terminus of HO-1 of rat origin	Antibody absorption with peptide on ICC	Rat postnatal day 6 cerebellum cortex*/neurons and blood vessels
HO-2/	1:1000/Rabbit	Rat, mouse, human, rabbit, cow, dog/full length protein	No antibody on ICC	Rat cerebellum*/Purkinje cell soma
Abcam iNOS/Chemicon	polyclonal 1:1000/rabbit polyclonal	Rat, mouse/C-terminal 14 aa peptide from mouse macrophage iNOS	No antibody on ICC	LPS mouse brain, inner ear*/macrophages in blood vessels
nNOS/Calbiochem	1:1000/rabbit polyclonal	Rat, mouse, human/peptide corresponding amino acids 724–739 of human brain NOS coupled to KHL	No antibody on ICC	Normal rat, mice, PCs and layer 5 primary neurons
eNOS/Chemicon	1:500/mouse monoclonal	Rat, mouse, bovine, human/Bovine eNOS purified from cultured aortic EC	No antibody on ICC	Blood vessels from human and mouse brain.
GFAP/Sigma	1:1000/Mouse monoclonal	Rat, pig, human/Purified GFAP from pig spinal cord	No antibody on ICC	Human cortex and cerebella.
Nitrotyrosine/Chemicon	1:1000/mouse polyclonal	Rat, mouse, human/nitrated KLH	No antibody on ICC	Normal human cerebellum** 95 year old/neuronal somata
GAD67/Chemicon	1:2000/mouse monoclonal	Rat, mouse, human/Recombinant GAD67 protein	No antibody on ICC	Normal mouse brain and cerebellum.
Ferritin H/Santa Cruz	1:500/rabbit polyclonal	Rat, mouse, human/aa 131–183 of ferritin heavy chain of human	No antibody on ICC	Normal human cerebellar cortex** 98 year old/neuronal somata
Ferritin L/Sigma	1:1000/rabbit polyclonal	Rat, mouse, human horse spleen ferritin	No antibody on ICC	Normal human cerebellar cortex** 98 year old/glial cells

* From rats in the present study, ** Tissue available at authors laboratory, aa: amino acid, ICC: immunocytochemistry; LPS: lipo-polysaccharide treated mice; P: postnatal day; *** as per vendor specifications.

For immunofluorescence, cryostat prepared sections from the rat cerebella were incubated at room temperature for 1 hour with a blocking solution containing 1% bovine serum albumin (Fraction V, Sigma, SLM) and 1% Triton X-100 (Sigma) diluted in PBS. Next, the solution was removed and the primary polyclonal or monoclonal antibody was applied to the tissue sections. Antibodies and dilutions used are listed in Table 1. Tissue sections were incubated in a humid chamber at 4°C for 48 hrs. At the end of the incubation, tissue sections were washed 3 times for 10 minutes each in PBS. The secondary antibodies against rabbit or mouse primary antibodies and labelled with Alexa 488 or 594 (diluted 1:1000 in PBS, Molecular Probes) were applied and incubated for 1 hour at room temperature in the dark. At the end of the incubation, tissue sections were washed with PBS (3 × 10 min), and mounted with Vectashield solution (Vector Labs).

Positive and negative controls are described in Table 1. For the negative controls, sections were incubated with all solutions except for the primary antibodies during the immunocytochemical procedure. In the negative controls no specific immunoreactivity was detected. Cerebellar cortex sections from young normal mice and rats were used as positive controls for neurofilament proteins, synapsin-1 and GFAP. Lung and kidney sections from non-CO exposed controls were examined with antibodies against HO-1, SOD-1 and iNOS as positive controls (Table 1).

### Light and fluorescent microscopic documentation for quantification

The distribution of the cellular markers (Table 1) was studied in sagittal sections of the rat cerebella, located lateral 0.10 mm to 1.4 mm [45]. The cerebellar cortex is a complex but uniform structure; however, the functions of the different regions depend on the afferent input and the targets of the afferent commands [31-36]. Therefore for quantitative analysis it is important to sample the cerebellar cortex in a systematic fashion. To avoid bias, quantitative immunocytochemical analyses was made consistently on micrographs obtained randomly from the cerebellar cortex in lobules V to VIII [31-34].

Sections were viewed and imaged (at 200×) with an Olympus BX51 fluorescent microscope (Olympus America Inc, NY, USA) equipped with an Olympus DP70 digital camera. To provide unbiased comparisons of the immunoreactive signal from a particular antibody between each specimen, all images were captured using strictly the same camera settings. Images were acquired using MicroSuite™ Five software (Olympus America Inc). All images were prepared using the Adobe Photoshop™ software program run in a Dell Precision 380 computer. This program was used to optimize brightness and contrast, but not to enhance or change the image content. Photomicrographs for each cellular marker shown in this study illustrate representative immunocytochemical staining from representative sections from three rats cerebella/group from each of 3 different CO-exposure experiments (n = 9).

For quantitative immunocytochemistry cerebellar cortex sections for each experimental group were (simultaneously) immunoreacted with the same batch of antibodies and buffers. Tissue sections were incubated for the same period of time and secondary antibodies were washed at the same time (for fluorescent stained slides) or the DAB-HRP reaction were stopped at the same time using a chronometer.

### Quantification

Quantification of immunoreactivity was assessed and measured objectively by 2 independent observers. Immunoreactivity (DAB-chromogen and fluorescent signal) was measured using the ImageJ software program (Version 1.42 k, National Institutes of Health, USA, . ImageJ is a public domain program). Quantification methods were performed as described by others [46-48] and adapted to our immunoreacted tissue sections from the cerebellar cortex. As outlined above immunoreactivity for each cellular marker was measured consistently in three regions of each section of the cerebellar cortex within lobules V and VIII. Measurements per bio-marker were made on 3 immunostained tissue sections obtained from 3 rat cerebella per group/experiment.

The area of the entire cerebellar cortex (molecular, Purkinje and granular) was selected with rectangular selection tools. The chromogen, or fluorescence intensity of the selected (fixed) area was measured with analyze function-measurement RGB to obtaining a mean value. Green (Alexa Fluor 484) and red (Alexa Fluor 596) measurements (values) were used for quantification. Diaminobenzidine (chromogen) stained images were inverted with the Edit tool and blue color values were used for quantification. A background intensity was determined in a small window located away from the immunoreactive signal and was subtracted from the chromogen or fluorescence intensity value of the immunoreacted structures.

### Statistical Analyses

Mean values of chromogen or fluorescent intensity were averaged and subjected to one way repeated measures ANOVA analysis. Comparisons were made between group D and A, D and B, and D vs. C. Values were considered statistically significant at p ≤ 0.05. The Sigma Stat 3.1 software program (Jandel Scientific, San Rafael, CA) was used for statistical analyses.

### RNA isolation for real time RT-PCR

At the end of CO or air exposure rat pups were killed (as described above), the cerebellum was separated from the brain, and cut in two halves (sagittal) that were immediately snap frozen in liquid nitrogen and stored at -80°C. Some cerebellar tissue from these animals was used for another study reported, in part, in an abstract [49].

Before RNA isolation, frozen cerebellum samples from each group were immersed in RNAlater-ICE stabilizing solution (Ambion Inc., Austin, TX) and retained at -20°C for 16 hrs before RNA extraction. Each sample was then transferred into a sterile polypropylene centrifuge tube containing 1 ml of TRIzol solution (Invitrogen, Carlsbad, CA). Samples were homogenized (by sonication) using a dis-membrator (Model 100, Fisher Scientific) for 10 sec at room temperature (RT), and incubated in the TRIzol solution for 5 minutes. Two hundred μl of chloroform were added to the sample and thereafter shaken vigorously. Each sample was incubated 5 minutes at RT and centrifuged at 12,000 g for 15 minutes at 4°C. Five hundred μl of isopropanol were added to the supernatant to precipitate RNA. Ten minutes later the sample was centrifuged at 12,000 g for 10 min at 4°C. The supernatant was discarded and the pellet resuspended in 100 μl diethylpyrocarbonate (DEPC)-treated water.

RNA was first treated with DNAse to eliminate any contaminating genomic DNA before the conversion of RNA to cDNA. In brief, RNA was placed into a 200 μl PCR tube to which the following solutions were added: 10 μl of 10× RDD buffer (Qiagene), 2.5 μl of RNAse-Free DNAse I (6.8 units, Qiagene), and nuclease-free H_2_O to a final volume of 100 μl. The tube was placed into a thermocycler (Model 9600, Perkin Elmer) and incubated at 25°C for 10 min. DNAse was removed during the cleanup procedure using the RNeasy MinElute cleanup protocol (Qiagene). The resulting purified and concentrated RNA was quantified by a Qubit™ fluorometer using the Quanti-T assay kit (Invitrogen). Total RNA integrity and size distribution were checked by denaturing agarose gel electrophoresis and SYBR staining.

### Retrotranscription and RT-PCR

Total RNA was reverse transcribed using oligo (dT) 12–18 primers and SUPERSCRIPT III (Superscript III RT, Invitrogen) in a final volume of 20 μl at 42°C for 50 minutes. Real-time PCR was performed using 1 μL of cDNA for each reaction (equivalent to 50 ng of total RNA), 12.5 μL of SYBR Green/ROX qPCR, master mix from SuperArray BioScience Corporation (Frederick, MD, USA), 10.5 μl of ddH_2_O and 1 μl of the following rat gene-specific primers: β-actin (Cat # PPR06570B), nNOS also called NOS1 (Cat # PPR44930E), iNOS also called NOS2 (Cat # PPR44835A), and eNOS also called NOS3 (Cat # PPR49724A) all from SuperArray (in a final volume of 25 μL). All experiments were performed in triplicate and the PCR reaction mixture was run in the Mx3000P Real Time PCR-System (Stratagene). The thermal cycling conditions included an initial denaturizing step at 95°C for 10 min, 40 cycles at 95°C for 10 s, 60°C for 20 s, and 72°C for 30 s, followed by melting curves to differentiate non-specific primer-dimmers and specific amplicon. Each cDNA sample was tested for target genes of interest and a housekeeping gene. To verify that the reagents were not contaminated with DNA, or that the primers do not produce a signal by dimmer formation, a no-template control sample was included for each primer set. Amplicons were analyzed by 4% agarose gel electrophoresis.

### Data analyses

The threshold cycle (CT) is defined as the PCR cycle number at which the fluorescence intensity crosses a manually determined threshold value, at a level where the fluorescent signal is appreciably above the background level but is still in the early exponential phase of amplification. Each of the 4 samples in each group was assayed in triplicate, the resulting CT values were averaged and the standard deviation calculated. If the standard deviation of the triplicate values was more than 0.3 then the value for that sample was not used in the final analysis. Next, for each pool of RNA the difference in the averaged CT values (CT-avg) for a gene of interest and β-actin for a given experimental group was calculated and defined as ΔCT for each gene of interest. The ΔCT value for each of four pools for each experimental group was then averaged and the standard deviation (S.D.) calculated. For each gene the average ΔCT value for the control group (group D), was subtracted from the average ΔCT value of the experimental groups (A, B, and C) resulting in the ΔΔCT. The error associated with ΔΔCT was equivalent to the standard deviation of the mean of the ΔCT for the experimental group. The data are reported as fold change, which was calculated as 2^-ΔΔCT^. This assumes doubling of products every amplification cycle or 100% amplification efficiency. The standard error is represented as 2^-(ΔΔCT ± SD)^. The significance of the ΔΔCT values was evaluated by performing a Student t-test using Sigma Stat software (Jandel Scientific, San Rafael, CA) with a p = 0.05 considered to be statistically significant.

## Results

### Superoxide dismutase-1 immunoreactivity (SOD-1-IR)

**Figure 1:**
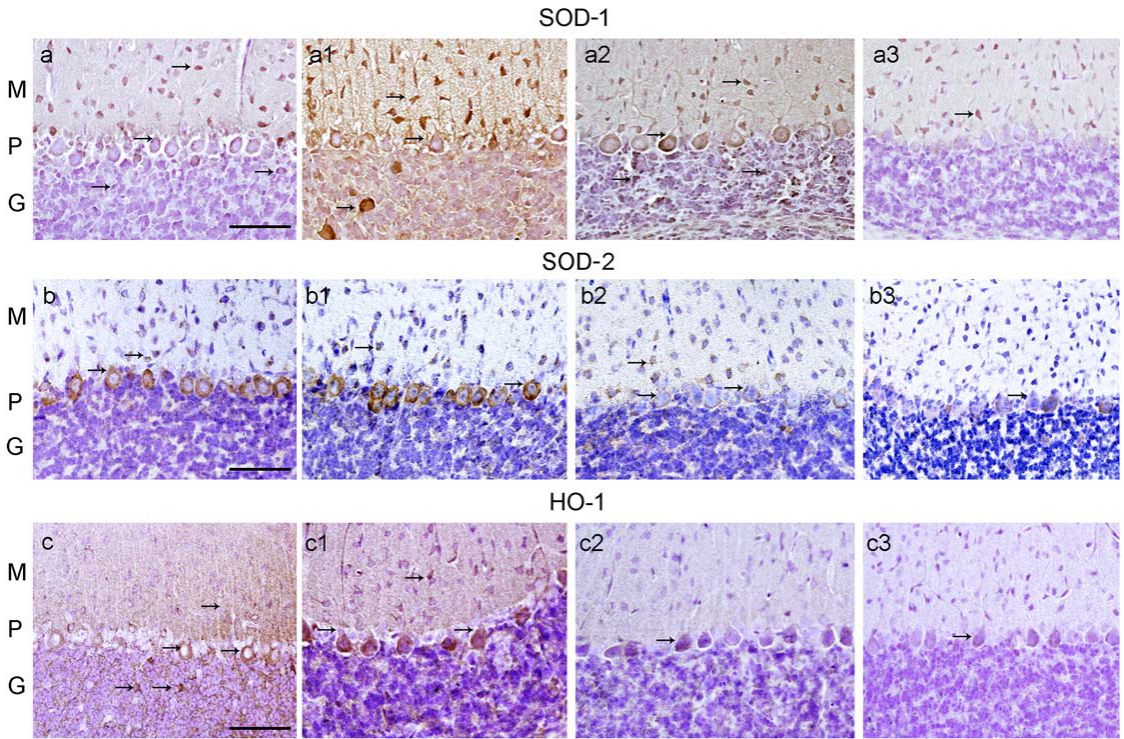
**SOD-1, SOD-2 and HO-1 IR in the cerebellar cortex of CO-exposed rats and their age matched controls**. Fig. (a), (a1) and (a2), SOD-1-IR in group A, B, and C respectively (appearing as an amber colored precipitate) was present in neurons of the molecular (M), Purkinje (P), and granular (G) layers (see arrows). (a3) group D shows normal SOD-1 IR. Fig. (b), and (b1), shows SOD-2-IR in neuronal bodies in M and P layers (see arrows). (b2) group C in contrast, showed less pronounced SOD-2-IR. (b3) group D shows normal SOD-2 IR. Fig. (c), and (c1), shows elevated HO-1-IR in neuronal bodies in M, P, and G layers (see arrows). (c2) group C, showed less pronounced HO-1-IR. (b3) control group D shows basal HO-1-IR. Hematoxylin (in purple color) identifies cell nuclei. Bar in all Figs. is 150 um.

SOD-1-IR was elevated in the cytoplasm of PCs, basket and granular cells in groups A, B and C (Fig. 1a, a1, and 1a2), especially in group B, when compared to the controls in group D (Fig. 1a3) where cells of the molecular layer showed a modest staining of SOD-1-IR. Quantitatively SOD-1-IR showed a significant increase in groups A, B and C with respect to group D (p ≤ 0.05) (Table 2).

**Table 2:** Immunocytochemical quantification in the cerebellar cortex of groups A, B, C and D.

**Marker**	**Group A**	**Group B**	**Group C**	**Group D**
**SOD1**	43.8 ± 1.2 (+24)*	73.6 ± 6 (+108)*	62.8 ± 1.5 (+78)*	35.3 ± 1.2
**SOD2**	62.8 ± 5.8 (+83)*	78.6 ± 6.7 (+129)*	36 ± 1.8 (+5)	34.2 ± 2.7
**HO1**	49.5 ± 2.6 (+115)*	54.2 ± 1.7 (+135)*	37 ± 1.4 (+61)*	23 ± 1.9
**iNOS**	53.2 ± 4.1 (+93)*	65.4 ± 5.3 (+138)*	59 ± 3.7 (+114)*	27.5 ± 2.5
**nNOS**	51.8 ± 6 (-13)	36.3 ± 4 (-61)*	52.3 ± 6 (-12)	59.2 ± 8
**eNOS**	79.5 ± 7 (+6)	80.3 ± 6 (+7)	73 ± 6 (-3)	75.3 ± 5
**Nitrotyrosine**	25.7 ± 4.3 (+109)*	31.8 ± 2.6 (+159)*	26.4 ± 3.9 (+115)*	12.3 ± 2.2
**Neurofilaments**	39.5 ± 4 (-61.71)*	35.6 ± 5.2 (-56)*	40 ± 6.2 (-62)*	64 ± 4
**GFAP**	64.6 ± 1.4 (+131)*	45.8 ± 1.4 (-7)	47.2 ± 1.9 (-4)	49.2 ± 2.2
**Parvalbumin**	44.5 ± 9.8 (-1.7)	37.8 ± 7.5 (-17)	44 ± 8.5 (-2.9)	45.3 ± 9.5
**GAD**	63 ± 5.2 (-20)*	60 ± 5.8 (-24)*	57.6 ± 4.6 (-27)*	79 ± 7.8
**Calbindin**	44 ± 12 (-10)	48 ± 12.5 (-2)	46.7 ± 12 (-5)	49 ± 13
**Synapsin**	31 ± 4 (-39)*	17.6 ± 3 (-65.5)*	42 ± 6.5 (-18)	51 ± 4.4
**Ferritin-H**	80 ± 5.6 (+1.3)	113 ± 5 (43)*	79.5 ± 1.9 (+0.6)	79 ± 3.6

± = standard error of the mean.

() = % from the control. + = increased immunoreactivity, - = decreased immunoreactivity.

Comparisons were made by one way ANOVA. Group D vs. group A, group D vs. group B and group D vs. group C. *Values were considered statistically significant when p values were ≤ 0.05.

nns: non significant.

### Superoxide dismutase-2 immunoreactivity (SOD-2-IR)

SOD-2-IR was elevated in PCs and basket cells in groups A and B (Fig. 1b and 1b1), when compared to group C and group D (Fig. 1b3) where cells of the molecular layer showed a modest staining of SOD-2-IR. Quantitatively SOD-2-IR showed a significant increase in groups A and B, but not in group C, with respect to group D (p ≤ 0.05) (Table 2).

### Heme oxygenase-1 immunoreactivity (HO-1-IR)

Figs. 1c and 1c1 shows that HO-1-IR was increased in PCs, basket and granular cells as well as the neuropile of the cerebellar cortex in CO exposed group A and B respectively. Less prominent HO-1-IR was observed in group-C (Table 2). HO-1-IR in the controls (group D) is minimal, (Fig. 1c3). Quantitatively HO-1-IR showed a significant increase in groups A, B and C with respect to the control in group D (p ≤ 0.05) (Table 2).

### Inducible (iNOS), neuronal (nNOS) and endothelial nitric oxide synthase (eNOS) immunoreactivity

**Figure 2:**
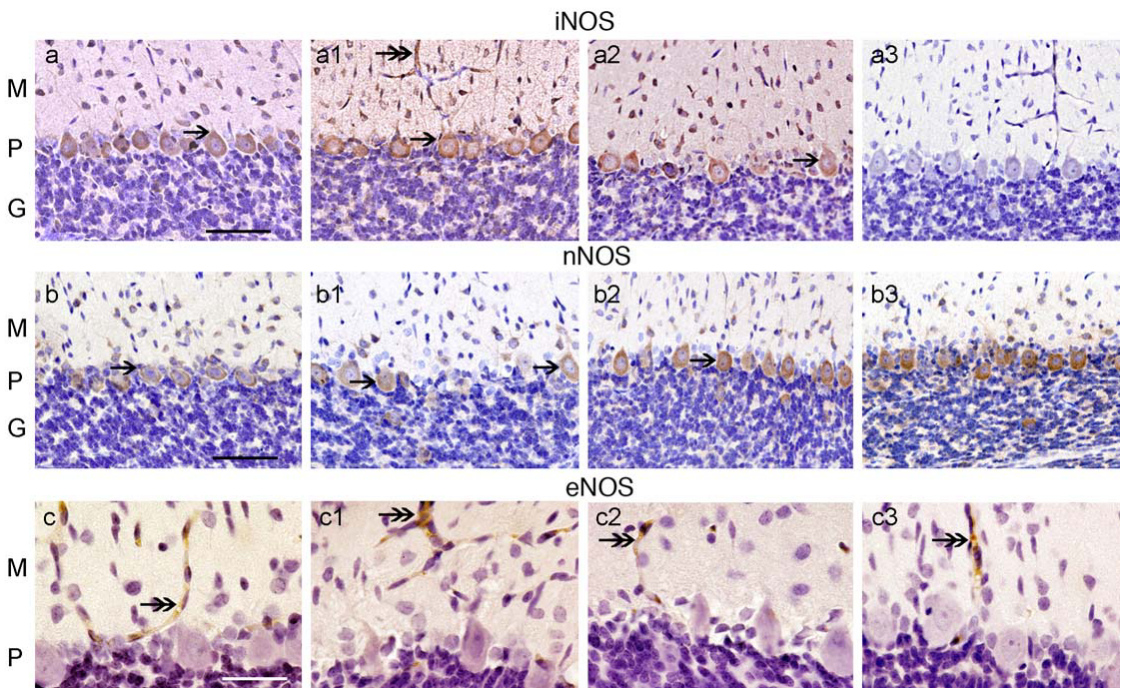
**iNOS, nNOS and eNOS – IR in the cerebellar cortex of CO-exposed rats**. Fig. (a), (a1), and (a2), shows elevated iNOS-IR (see arrows), in neurons of the 3 layers in group A, B and C. (a3), group D shows almost no iNOS IR. Fig. (b, b1, b2, and b3) shows nNOS-IR mainly in neuronal bodies in the P layer (see arrows). Group B shows decreased nNOS-IR in PCs, group A and C shows close to normal nNOS IR. (b3) group D shows normal nNOS-IR. Fig. (c), (c1), and (c2), shows eNOS-IR in blood vessels in groups A, B, and C, respectively. eNOS IR in the 3 CO-groups was similar and close to normal. (c3) group D shows normal eNOS IR. Hematoxylin (in purple color) identifies cell nuclei. Bar in Fig. a-a3, and b-b3 is 150 um. Fig. c-c3 bar is 35 um.

There was a significant increase of iNOS-IR in the soma of the Purkinje and basket cells of the cerebellar cortex of groups A, B and C (Fig. 2a, a1 and 2a2 respectively) when compared to group D (Fig 2a3). Inducible-NOS-IR was also present in endothelial cells of the blood vessels (Fig. 2a, a1 and 2a2). Quantitatively iNOS-1-IR showed a significant increase in groups A, B and C with respect to the control in group D (p ≤ 0.05) (Table 2).

Neuronal-NOS-IR was reduced in PCs of group B (Fig. 2b1), with less significant changes in groups A and C (Fig 2b and 2b2, respectively), when compared with group D (Fig. 2b3). Quantitatively nNOS-IR showed a significant decrease in group B with respect to group D (p ≤ 0.05) (Table 2), however, no significant changes were seen in nNOS-IR in-group A and C.

Endothelial-NOS-IR was present in blood vessels of the cerebellar cortex and remained unchanged in all CO-groups (Fig. 2c, c1 and 2c2) when compared to group D (Fig. 2c3). Quantitatively eNOS-IR showed no significant changes in groups A, B and C with respect to group D (Table 2).

### NOS mRNA expression

**Figure 3:**
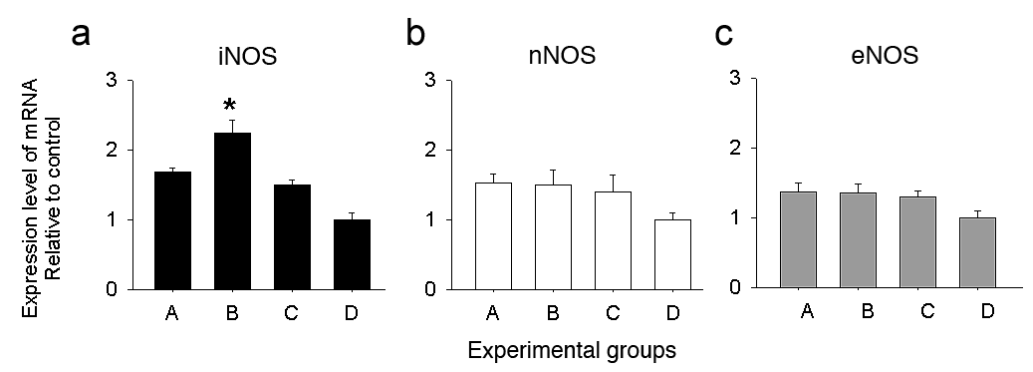
**Quantitative real time RT-PCR expression of iNOS, nNOS and eNOS in the cerebellar cortex of CO-exposed rats**. Fig. (a) iNOS mRNA was significantly up-regulated (asterisk) in group B, followed by groups A and C, group D shows the normal expression. (b) nNOS and (c) eNOS mRNA in groups A, B and C, showed no significant changes with respect to control group-D. Results represent standard error of the mean from 3 experiments for each group.

Gene expression levels for iNOS, nNOS and eNOS were determined by real time PCR using SYBG and the data normalized according to the level of β-actin mRNA, a non-regulated reference gene (Fig. 3). Data were expressed as fold change where the data for the group D, the reference, was assigned 1 fold (no change or normative value). Graphs in fig. 3a, b and 3c show a comparison of the three CO-exposure groups A, B and C with the control group D. There was a significant increase in the expression of iNOS mRNA in the cerebella from group B. INOS mRNA expression also increases in groups A and C, however, this increase was less significant than group B. Neuronal-NOS and eNOS showed a less than 1 fold change in mRNA expression in all three CO groups with respect to the control.

### Nitrotyrosine immunoreactivity (NT-IR)

**Figure 4:**
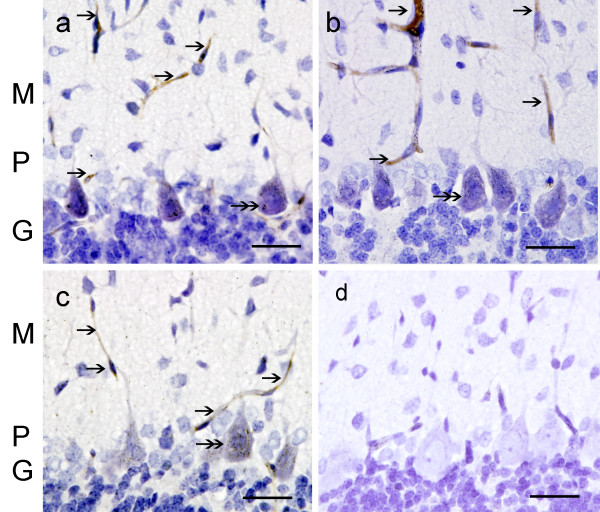
**NT-IR in the cerebellar cortex of CO-exposed rats**. Fig. (a) group A, (b) group B, and (c) group C, shows NT-IR in blood vessels (single arrows) of the 3 layers. PC cytoplasm showed NT-IR (double arrows). Fig. (d) group D, NT-IR was absent in blood vessels or neurons. Hematoxylin (in blue-purple color) identifies cell nuclei. Bar in all Figs. is 75 um.

NT-IR was found in blood vessels of the molecular layer in groups A, B and C (Fig. 4a, b, and 4c). PC bodies showed NT-IR to a lesser degree. NT-IR was not detected in controls (Fig. 4d). Quantitatively NT-IR was significantly increased in all CO groups with respect to group D (p ≤ 0.05) (Table 2).

### Neurofilaments (NF) and glial fibrilar acidic protein (GFAP) immunoreactivity (IR)

**Figure 5:**
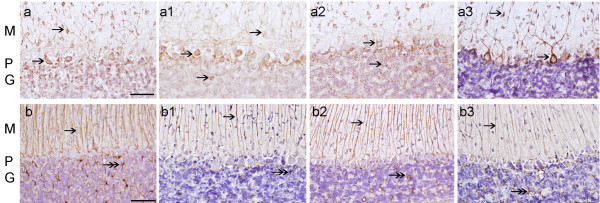
**NF-IR and GFAP-IR in the cerebellar cortex of CO-exposed rats**. Fig. (a) group A, (a1) group B, and (a2) group C, showed decreased NF-IR in neurons of the 3 layers (arrows). (a3) group D shows normal NF-IR. (b) Group A, shows higher GFAP-IR in glial cells in M, P, and G layers (see arrows) than group D (b3). (b1) and (b2) group B and C in contrast, showed close to normal GFAP-IR. (b3) group D shows basal GFAP-IR. Hematoxylin (in purple color) identifies cell nuclei. Bar in all Figs. is 150 um.

Decreased NF-IR was observed in groups A, B and C. Specifically NF-IR was decreased in the cytoplasm of PCs, basket cells and granular cells (Fig. 5a, a1 and 5a2) when compared with group D (Fig. 5a3). Quantitatively NF-IR was significantly decreased in all CO groups with respect to group D (p ≤ 0.05) (Table 2). GFAP-IR was increased in glial cells of the cerebellar cortex of group A (Fig. 5b1). GFAP-IR in-groups B and C (Fig. 5b1 and 5b2) were similar to group D (Fig. 5b3). GFAP-IR was significantly increased in-group A, but not in groups B and C (p ≤ 0.05) (Table 2).

### Parvalbumin (PV), GAD, calbindin (CB), and Synapsin-1 immunoreactivity

**Figure 6:**
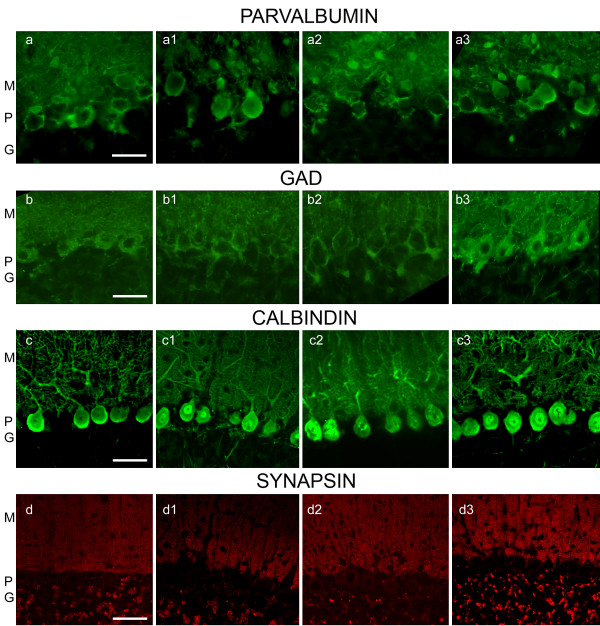
**PV, GAD, CB and Syn-1-IR in the cerebellar cortex of CO exposed and control rat pups**. Fig. (a), (a1), (a2) and (a3), PV IR in groups A, B, C, and D respectively shows PV-IR (in green color) in neuronal bodies and processes in the M, P, and G layers. PV-IR in all 3 CO-groups was not different when compared to group D. Fig. (b), (b1), (b2) and (b3), GAD-IR in groups A, B, C, and D respectively shows GAD-IR (green color) in neuronal bodies, processes and button terminals in the M, P, and G layers. GAD-IR in all 3 CO-groups was decreased when compared to the control, group D. Fig. (c), (c1), (c2) and (c3), shows CB-IR in groups A, B, C, and D respectively. CB-IR (green color) is localized mainly in PCs and their processes. Fig. (d), (d1), (d2) and (d3), shows Syn-1-IR (red dots) in groups A, B, C, and D. Syn-1-IR is localized in button terminals respectively of the 3 layers. There was a pronounced decrease in Syn-1-IR in group A and B and to a lesser extent (but not significant) in group C. Scale bar is 150 um in Figs. a-a3, and b-b3, and 200 um in figs c-c3 and d-d3.

PV-IR in all CO-exposed groups (Fig. 6a, a1 and 6a2) was similar to the control group (Fig. 6a3). There were no significant quantitative changes in PV-IR between groups (Table 2). In contrast GAD-IR was reduced in the soma of PCs, and in the cells in the molecular layer of CO exposed rat groups A, B and C (Fig. 6b, b1 and 6b2) when compared to group D (Fig. 6b3). Quantitatively GAD-IR was significantly decreased in groups A, B and C with respect to group D (p ≤ 0.05) (Table 2). CB-IR remains unchanged in groups A, B and C (Fig. 6c, c1 and 6c2) with respect to the controls (Fig. 6c3). There were no significant quantitative changes in CB-IR among groups (Table 2). Synapsin-1-IR was decreased significantly in nerve terminals in the molecular layer in-group A and B (Fig. 6c and 6c1) and almost unchanged in-group C (Fig. 6c2) with respect to group D (Fig. 6c3). Quantitatively, synapsin-IR significantly decreased in nerve terminals in-group A and B, but not in-group C (p ≤ 0.05) (Table 2).

### Ferritin-H immunoreactivity (FH-IR)

**Figure 7:**
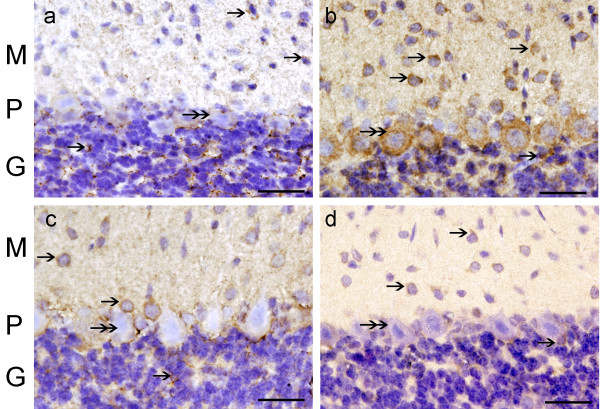
**FH-IR in the cerebellar cortex of CO-exposed rats**. Fig. (a) group A, (b) group B, (c) group C and (d) group D, shows FH-IR appearing as an amber colored precipitate, (see arrows) in neuronal bodies and neuropile in the M, P, and G layers. Hematoxylin (in purple color) identifies cell nuclei. Bar in all Figs. is 150 um.

FH-IR was increased in PCs of group B (Fig. 7b1), with less significant changes in groups A and C (Fig. 7a and 7c, respectively), when compared with FH-IR in group D (Fig. 7b3). Quantitatively FH-IR was significantly increased in group B with respect to group D (p ≤ 0.05) (Table 2). No significant changes in FH-IR was seen in group A and C, however, there seems to be a redistribution of immunoreactivity (punctated) pattern in cells of the granular layer in group A, as compared with group D.

## Discussion

In this study we found that chronic mild CO exposure (25 ppm) promotes an increase in SOD-1, SOD-2, HO-1, iNOS and NT in the cerebellum. These changes correlated with a decrease in neurofilament proteins, synapsin-1 and GAD immunoreactivity. The cerebellar cortex from group B (pre and postnatal CO-exposure) showed a similar pattern of immunoreactivity as for group A (prenatal CO exposure). Immunocytochemical changes were less prominent in group C (postnatal exposure). Changes with these markers and treatments were studied only at P20, when the development of the cerebellum is yet active. These results suggest that deleterious and protective mechanisms were activated upon mild chronic CO exposure over the period of dynamic development of the cerebellar cortex.

Prenatal CO exposure in our rat model simulates the conditions of CO exposure of a human fetus whose mother is a mild to modest cigarette smoker, with clinical implications, in terms of persistent/permanent damage to fetuses caused by CO in cigarette smoke or from the ambient air that contains CO. Tobacco smoking during pregnancy has been implicated as an etiologic factor of low birth weight and pre-maturity, *in utero; *cigarette smoke exposure restricts blood flow in the uterine and umbilical-placental circulation either acutely through nicotine and/or other vasoactive agents released in response to smoking, or chronically by structural alterations to the placenta [50]. In our most recent publication on the rat peripheral auditory system [42], we showed that prenatal chronic mild CO exposure at the same concentration used in the present study increases SOD-1, HO-1, iNOS and NT immunoreactivity parallel to deterioration of afferent terminals that innervate the inner hair cells within the developing cochlea [42].

### Oxidative stress and damage

Oxidative stress in a physiological setting can be defined as an excessive bioavailability of reactive oxygen species (ROS), which is the net result of an imbalance between production and destruction of ROS (with the latter being influenced by antioxidant defences) [51]. The contemporary definition of oxidative stress has been refined to account for two different mechanistic outcomes, macromolecular (oxidative) damage, and disruption of thiol redox circuits, which leads to aberrant cell signaling and dysfunctional redox control [51,52]. Macromolecular damage is considered in terms of oxidative mechanisms linked to free radicals, which are small, diffusible molecules that differ from most biological molecules in that they have an unpaired electron (i.e. superoxide anion radical, CO, NO, hydroxyl radical). The free radicals NO and superoxide react to generate the non-radical oxidant peroxynitrite. Thiol-containing proteins that function in redox signaling and physiological regulation are susceptible to two-electron oxidation by non-radical oxidants, including hydrogen peroxide, lipid peroxides, aldehydes, quinines and disulfides [53]. Either abnormal oxidation or irreversible modification can interfere with reversible oxidation-reduction reactions of thiols that physiologically function in receptor signaling, transcriptional regulation, cell proliferation, angiogenesis and apoptosis. Thus the contemporary refinement in the definition of oxidative stress represents a shift to include both non-radical oxidants and reversible oxidative reactions of redox signaling and controls as key components of oxidative stress.

### CO in cellular redox signalling

As reviewed by Piantadosi et al., and references therein [54], cellular effects of CO are produced primarily by CO binding to iron or other transition metals, which may also promote pro-oxidant activities of the more reactive gases, oxygen and NO. The effects of CO on the mitochondrial oxidation-reduction (redox) state are well recognized because CO binds the reduced iron (Fe^2+^) in the *a*_3 _heme of the terminal electron acceptor of the respiratory chain, cytochrome c oxidase. CO increases mitochondrial ROS production in vitro and in vivo, and mitochondria metabolize CO by oxygenation to CO_2_. CO also binds to reduced transition metals and other metalloenzymes including guanylate cyclase (GC) and cytochrome P_450_, therefore, CO-heme ligand formation tends to interfere with redox reactions involving molecular oxygen as well as NO. The activation of the GC system by CO and NO occurs by binding the active heme and enhancing cGMP production [54]. NO is more potent than CO in activating GC in vasodilation, CO regulates vascular smooth muscle in hypoxia. NO unlike CO, binds to both Fe^2+ ^and Fe^3+ ^heme, and low CO concentrations stimulate NO release and are associated with peroxynitrite generation in vascular cells [2,5]. It has been proposed by Calabrese et al., [55] that HO-1 and/or CO and iNOS and/or NO are functionally interrelated in mediating their protective effects. In some situations, CO can activate the expression of iNOS, in others, inhibits the expression of iNOS. NO up-regulates HO-1 with the production of CO [55].

### SOD-1 and SOD2

SOD-1 immunoreactivity was high in PCs of the cerebellar cortex in groups A, B, and C suggesting that SOD-1 and SOD-2 may serve as a defense system against ROS induced injury. SOD-1 has a widespread distribution in a variety of cells [56]. SOD-1 is constitutively expressed, and its mRNA levels can be dramatically upregulated by a wide array of mechanical, chemical and biological messengers. SOD-2 is a highly inducible mitochondrial protein capable of protecting the brain from oxidative damage; its expression has been demonstrated to be critical in modulating the response of neurons to different type of stress [56,57]. SOD-2 is synthesized within the cytosol and imported to the mitochondrial matrix, where it converts superoxide anion to hydrogen peroxide. Within the mitochondria, the hydrogen peroxide is then converted either to water by mitochondrial glutathione peroxidase or catalase, or can participate in Fenton type chemistry, giving rise to further ROS such as the hydroxyl radical [58]. Thus, SOD-2 can acts as a fundamental defense against ROS within the mitochondria [59]. Increased levels of SOD-2 protein, mRNA, and enzymatic activity have been amply documented in the rodent brain as a consequence of normal aging, ischemia, hyperoxia, and pro-oxidant drug exposure [58-60]. We detected a significant increase in SOD-2-IR in cerebellar neurons in groups A and B, and a less significant change in group C. These results suggest that a protective mechanism mediated by SOD-2 is activated after CO exposure.

### HO-1 and HO-2

HO-1 but not HO-2 was up regulated in neurons of the cerebellar cortex of CO exposed pups in groups A and B, and in contrast, there was a mild upregulation of HO-1 in group C. The heme oxygenase isozymes, HO-1 and HO-2, oxidatively cleave the heme molecule to produce biliverdin and CO [61-66]. The cleavage results in the release of iron, a regulator of transferrin, ferritin and NOS gene expression [61,65]. HO-1 is a heat shock protein (HSP-32) induced under numerous conditions of cellular stress [63-66]. HO-1 upregulation suggests that a protective mechanism is activated in response to adverse cellular oxidative conditions. HO-2-IR did not change in the three CO exposed groups with respect to the control (not shown). HO-2 is responsible for most of the HO constitutive activity [64].

### Nitric oxide synthases and NT in the cerebellar vasculature after CO exposure

There was a significant upregulation of iNOS in the soma of the PCs and endothelial cells of the blood vessels within the cerebellum in all CO-exposed groups. The upregulation of the iNOS is accompanied by an increase in the expression of iNOS mRNA. Evidence that the upregulation of iNOS produces a detrimental amount of its product, NO, in the cerebellum was shown by the presence of NT in endothelial cells of the blood vessels and to a lesser extent the soma of the PCs. NT is a relatively specific stable biochemical marker for peroxynitrite formation [67] particularly when significant amounts are present [68-70]. Inducible-NOS is also controlled by inflammatory mediators and cytokines and iNOS up-regulation, produces large quantities of NO, whereas nNOS and eNOS produce low amounts of NO [71]. Exogenous and endogenous CO exerts its action by modifying the NO system, including modulation of soluble guanylate cyclase [72-75]. Our results suggest that chronic exposure at mild CO concentrations (12–25 ppm) perturb both the CO and NO systems in the cerebella. The persistence of markers indicating oxidative stress in group A by P20, following prenatal only CO-exposure demonstrates the condition is sustained.

By contrast, nNOS-IR was substantially reduced in the cerebellum of CO-exposed animals from group B, with no significant change in groups A and C. A decrease in nNOS-IR without a detectable change in the abundance of the mRNA, suggests that oxidative stress may affect the function of this enzyme [76]. Interestingly eNOS in the three CO-exposed groups remains relatively unchanged with respect to the control. It is possible that with time (i.e. more than 20 days) after CO exposure a change in eNOS expression may become evident.

We only detected NT as an index of protein nitrosylation, additional methods such as protein oxidation [77] could supplement our findings. Also it is important to mention that the detection of lipid peroxidation and DNA oxidation have yet to be pursued in our laboratory to provide the definitive account on whether the upregulation of the several enzymes examined prevent oxidative damage or not. There is also a need to determine whether these changes are permanent or transient by studying their expression in adult animals following our prenatal/postnatal CO-exposure conditions.

### Prenatal CO exposure and hypoxia

When inhaled, CO is readily absorbed from the lungs into the blood-stream, where it forms a tight but slowly reversible complex with hemoglobin (Hb) known as carboxyhemoglobin (COHb). The presence of COHb in the blood decreases the oxygen carrying capacity, reducing the availability of oxygen to body tissues and resulting in tissue hypoxia [1,8]. At 10 ppm the estimated COHb in blood is 2%, and at 70 ppm it is 10% [1]. Using similar CO exposure conditions at 100 ppm on rat pups over the postnatal weeks we have reported COHb in amounts up to 10%, but there was some overlap with controls [39]. We did attempt to measure COHb in the animals exposed at 25 ppm, and at this concentration there was no obvious difference where values were in the range 2 to 5% for test and controls.

Carbon monoxide from tobacco smoke easily crosses the placental barrier by passive diffusion, causing a 4-fold increased level of COHb in umbilical cord blood; this has the property of inhibiting the release of oxygen into fetal tissues across the placenta in prenatal exposure [78]. The consequent chronic hypoxia may alter the physiological development of organs susceptible to hypoxic damage including the brain. Our data shows that prenatal CO exposure increases several proteins related to oxidative stress and neuronal function in a similar fashion that was found for prenatal plus postnatal exposure. Lavezzi et al., [78] showed a decreased noradrenergic activity in the locus coeruleus and EN2 gene expression in the arcuata nucleus and somatostatin in the hypoglossal nucleus in the brainstem by comparing still births of mothers who were smokers versus those from mothers who were non-smokers. In a related study Matturri et al., [79] found a significant relationship between maternal smoking and brainstem alterations (hypodevelopment of the arcuate nucleus, gliosis, hypoplasia of the hypoglossus nucleus).

The hypoxic effect by CO could be discerned by subjecting the animals to hypoxic only conditions [80] i.e. low oxygen concentrations (hypoxygenation), and then comparing the similar oxidative stress markers. Gene expression analyses could provide additional information. Gess et al., [80] subjected rats to hypoxia (low oxygen tension) or 0.1% CO (1000 ppm) from 0.5 to 6 hrs. They detected increased c-fos mRNA levels in the heart after 0.5 hr, and at 0.1% of CO after 6 hours of exposure the increase in mRNA was even more significant. There was an organ selective increase of c-fos, however they did not measure the levels of c-fos mRNA in the brain. We have begun to address this issue in our CO exposure model (25 ppm exposure) by screening for the expression of 86 hypoxia-associated genes in the rat cerebella using real time RT-PCR. We found up-regulation of erythropoietin and down-regulation of leptin mRNA with no significant changes on HIF factor 1 and 3 [49].

### Neurofilaments and synapsin after CO exposure

At P20, neurofilament and synapsin-1-IR are low in the three CO-exposed groups as compared to controls. A decrease in neurofilament proteins and synapsin-1, a marker for efferent terminals, and well described for the cerebellum [81,82] suggests that PCs and their terminals are very sensitive to chronic mild CO exposure. Our earlier studies on postnatal CO-exposure showed that chronic mild CO exposure permanently decreases basal c-fos in another region of the brain, the central nucleus of the inferior colliculus [40,41], a condition that can be prevented by the dietary reduction of iron without anaemia [41]. In this study we concluded the Fenton reaction was diminished sufficient to avoid the consequences of chronic mild CO exposure [41].

### Parvalbumin and Calbindin after CO exposure

We found that PV and CB immunoreactivity remain unaltered in the three CO-exposed groups. PV and CB are two calcium-binding proteins expressed abundantly in the cerebellar cortex [35,83-86]. The cerebellum is a highly organized structure in which PCs are the sole output of the cerebellar cortex. These cells are aligned in a single plane, parallel to the surface of the cortex and they can be specifically stained with antibodies directed against PV or CB [35,85]. In most species, all PCs (soma and dendrites) show CB-IR from an early stage of embryonic development [84,86]. It is suggested that calcium-binding proteins play a role in the regulation of intracellular calcium signaling cascades, therefore, important to the cell firing, and they are also related to changes in sensory inputs [83,86]. The fact that we found no changes in PV-IR or CB-IR does not rule out changes in these two proteins in the long term, or changes in other calcium binding proteins/molecules, such as calretinin or IP3.

### Glutamic acid decarboxylase

We found that CO exposure results in a significant decrease in GAD in all three CO exposed groups. GAD is the enzyme responsible for the synthesis of GABA, an important inhibitory neurotransmitter of the cerebellum, which contains a high number of GABA synthesizing neurons [87]. Our results are in line with previous studies that showed significant decreases in total cerebellar GABA content following pre- and postnatal exposure to 150 or 300 ppm of CO [29]. More recently two studies by Benagiano et al., [22,23] also demonstrate that prenatal CO exposure (75 ppm) affects (decreases) quantitatively GAD and GABA immunoreactivity in the rat cerebellar cortex. These studies were made in the cerebellar cortex of the Vermis. For these experiment [22,23], pregnant rats were exposed to 75 ppm CO in air from day 0–5 to day 20 of pregnancy. The resulting progeny were killed at 120–150 days postnatal. The 75 ppm CO-exposure condition used by Benagiano [22,23] induces a relatively mild decrease of oxygen availability to fetuses, but does not impair CNS development [88]. The decrease of GAD immunoreactivity in our study after prenatal, pre- and postnatal and postnatal CO exposure at 25 ppm, support the idea that lower concentrations of CO are still detrimental to cerebellar development and in turn would create physiological deficits.

### GFAP

In group A (prenatal only CO exposure), there is an up-regulation of GFAP-IR in astroglia in the granular layer and in the Bergman glial (BG) cells located in the PC and molecular layer [89,90]. In contrast, group B (pre and postnatal CO exposure) and group C (postnatal CO exposure only) show no alteration in GFAP-IR, being indistinguishable from data for the cerebella of group D. These results suggest that it is important to prevent CO exposure (even at very mild CO exposure) in the prenatal period. Either down-regulation or up-regulation of GFAP may have detrimental consequences to the proper development of the CNS [91]. Up-regulation of GFAP-IR indicated glial activation [92]. Indeed glial activation has been detected in the rat brain after acute CO exposure [92], and it has been suggested that activated microglia can mediate cognitive dysfunction by impairing neurogenesis and by causing necrosis or apoptosis [93,94].

### Ferritin

The iron-storage protein ferritin is a 24-subunit protein involved in the regulation of iron availability [95,96]. Mammalian ferritin contains variable amounts of two types of polypeptide chain, heavy (H) and light (L). H-chain ferritins are associated with responses to stress, whereas L-rich ferritins are associated with iron storage [97]. The subunit composition of ferritin proteins (H:L ratio) is cell- and tissue-specific. In the CNS, microglia contain mostly L-rich ferritin, neurons contain mostly H-rich ferritin, and oligodendrocytes contain equal amounts of both isoforms [98]. In the present study ferritin H was significantly increased in cells of the cerebellar cortex in group B (pre and postnatal exposure); there was a redistribution of FH-IR in cells of the granular layer in group A. Group C showed a similar immunoreactive patterns to the control group D. No changes in ferritin-L immunoreactivity were detected in cerebellar cells of the three CO exposed groups with respect to the air controls (not shown). Ferritin-H upregulation (increased immunoreactivity) may occur as a compensatory mechanism to counteract increased amounts of iron that is caused by the release of iron from heme proteins [95]. In this respect we did not detect free iron (using Prussian blue staining) in the rat cerebellar cortex of the three CO groups (data not shown).

### Relevance of mild chronic CO exposure during prenatal development

Our results show that the detrimental effect of chronic mild prenatal exposure to CO (at the levels expected from mild active smoking, second hand smoke in poorly ventilated home environments) can occur within the cerebella. This is important given that CO is not the only component in cigarette smoke, or the only source of this gas in the atmosphere [1]. The fact that markers for regulatory signal systems involving SOD-1, SOD-2, HO-1, iNOS, NT and ferritin are increased supports the idea that neuronal competencies are affected because of a persistent state of oxidative stress after chronic mild exposure to CO.

We propose that CO is not a toxin at low concentrations (0.0025% in air). Rather, a chronic environment of exogenously available CO can act as an aberrant neuromodulator *in-vivo*, subverting the signal activities of naturally produced CO and NO and related processes. This finding indicates that neuronal cell functions that depend on nNOS involvement are compromised in cells when there is prolonged NO production from at least the persistent over expression of iNOS. As a consequence, regulatory signal systems involving inducible HO-1 and iNOS are persistently not optimal.

Molecular and cellular effects of CO exposure, oxidative stress and redox signalling are well documented in several in-vivo and in-vitro models [2,3,5,54,55,99]. The changes we observed by immunocytochemistry and mRNA analyses on oxidative stress markers and on structural and functional proteins after prenatal chronic very mild CO exposure serve as the basis to examine mechanistic relationships among cell signalling pathways, antioxidant defense processes and putative pathological endpoints for the developing cerebellum.

The study of the effects of the chronic presence of very mild CO concentrations in air during prenatal development is relevant for several reasons: (a) *Vulnerability*. CO readily crosses the placenta, favoring a mild increase in oxidative stress that "normally" occurs in early development. Ferriero [100] indicates that the increased susceptibility to oxidative injury can be explained by several mechanisms that facilitate the production of reactive oxygen species from the increased presence of hydrogen peroxide that tends to accumulate because of immature defense mechanisms. It is indicated the inadequate scavenging abilities inherent to developing systems are not sufficient to maintain glutathione stores in neonatal neural tissue that also contains more available iron than is present in mature neural tissue. (b)*CO exposure conditions and the developing cerebella*. The rat cerebellar PCs are generated prenatal, whereas stellate or basket cells are produced postnatal [101]. Thus the 3 CO exposure regimens showed a differential expression of the cellular markers examined in the present study. The combination of these circumstances in normal development can result in a mild state of oxidative stress [100]. (c)*Relevance of mild CO concentrations*. The chronic mild CO exposures used in our studies, (intermittent and averaging about 12 hours in each 24 hour period) are not toxic (poisonous) but at amounts below the upper limits identified by most regulatory agencies as previously indicated [41]. The exposures are the same or less than that reported for the home environment especially where gas appliances are used, and below the concentrations indicated to prevail in active tobacco smokers who are pregnant or breast feed their offspring. For example, no standards for CO exposure have been agreed upon for indoor air and the EPA indicates that average CO levels in homes without gas stoves vary from 0.5 to 5 ppm, (5 ppm is 0.0005% CO in air). Levels near properly adjusted gas stoves are often 5 to 15 ppm and those near poorly adjusted stoves may be 30 ppm or higher. It is also indicated that today there is an emphasis on insulating homes to save energy, conditions that can contribute to poor ventilation . (d) *Significance to existing human conditions*. Today oxidative stress is recognized as a condition common to many health problems [99,102,103]. It is considered to be a factor in the ageing process and known to be involved in the initiation of cardiovascular disease. Oxidative stress has been shown to be a component of many neurodegenerative diseases such as Alzheimer's, Parkinson's, Huntington's, amyotrophic lateral sclerosis and multiple sclerosis, as examples. A search of the scientific literature on oxidative stress and for example, asthma, cancer, and autism reveals that conditions of oxidative stress can prevail. In our animal model we have shown CO exposure can create and promote conditions of oxidative stress. In the human situation where oxidative stress is already present it can be predicted that chronically supplied CO from the air or from the active smoking of tobacco can be expected to exacerbate any of these health conditions leading to a more pronounced condition of oxidative stress.

## Conclusion

Six distinctive markers (HO-1, SOD-1, SOD2, ferritin, iNOS and NT), known to be increased when there is oxidative stress were significantly increased by exposure to chronic mild CO concentrations over the fetal period and the early postnatal period. Further, the up-regulation of the gene for iNOS in relation to the generation of ROS corroborated the immunocytochemical observations. The persistence of markers indicating oxidative stress at 20 days postnatal following prenatal only CO-exposure demonstrates the condition is chronic. The outcome from a persistent oxidative stress may be selective. Synapsin-1, neurofilament proteins and GAD, were significantly decreased suggesting a suboptimal expression that would not meet their respective cellular functions. The detrimental effect of chronic mild CO exposure on the cerebellar cortex may be reflected in balance deficits given that an adequate neural innervation is essential for the normal development of neurons in the cerebella.

## Abbreviations

CB: Calbindin-D28K; CO: carbon monoxide; Heme oxygenase-1: HO-1; HSP-32: heat shock protein-32; SOD-1: superoxide dismutase-1; SOD-2: superoxide dismutase-2; NO: nitric oxide; iNOS: inducible nitric oxide synthase; nNOS: neuronal nitric oxide synthase; eNOS: endothelial nitric oxide synthase; GAD: glutamic acid decarboxylase; GFAP: glial fibrillary acidic protein; NT: nitrotyrosine; IR: immunoreactivity; ferritin-H: FH; PBS: phosphate buffered saline; ROS: reactive oxygen species; PV: parvalbumin; PCs: Purkinje cells; CNS: central nervous system; EPA: Environmental Protection Agency; ppm: parts per million; E5: embryonic day 5; P20: postnatal day 20.

## Competing interests

The authors declare that they have no competing interests.

## Authors' contributions

IAL and JE conceived, designed the experiments, wrote and edited the manuscript. DA provided imaging data and performed immunocytochemistry. LB developed and performed real time PCR experiments. IEL, AA, MC carried out immunofluorescence staining and captured images. All authors observed immunoreactivity on the cerebellar sections for qualitative analysis, read and approved the final manuscript.
